# 8-Methyl-3-methyl­sulfanyl-8a,8b-di­hydro-5*H*-1-oxa-2,4-di­aza­ace­naphthyl­ene

**DOI:** 10.1107/S241431462100674X

**Published:** 2021-06-30

**Authors:** Akoun Abou, Fanté Bamba, Jérôme Marrot, Soro Yaya, Jean-Marie Coustard

**Affiliations:** a Department of Training and Research in Electrical and Electronic Engineering, Research Team: Instrumentation, Image and Spectroscopy, Félix Houphouët-Boigny National Polytechnic Institute, BP 1093 Yamoussoukro, Côte d’Ivoire; b Laboratoire de Constitution et Réaction de la Matière, UFR SSMT, Université Félix Houphouët-Boigny, 22 BP 582 Abidjan 22, Côte d’Ivoire; c Laboratoire ILV-UVSQ-UMR 8180 CNRS, 45 Avenue des Etats Unis, 78035 Versailles Cedex, France; d Laboratoire des Procédés Industriels de Synthèse et d’Environnement, Institut National Polytechnique Félix Houphouët-Boigny, BP 991 Yamoussoukro, Côte d’Ivoire; e Laboratoire IC2MP-UMR 7285 CNRS, 40 Avenue du Recteur Pineau, 86022 Poitiers Cedex, France; Zhejiang University (Yuquan Campus), China

**Keywords:** crystal structure, di­aza­dihydro­ace­naphthyl­ene derivative, hydrogen bonding and C—H⋯π inter­actions

## Abstract

In the tricyclic title compound, C_11_H_12_N_2_OS, the 2,3,4,5-tetra­hydro­pyridine ring adopts a half-chair conformation. This ring makes dihedral angles of 27.72 (7) and 45.17 (7)°, respectively, with the isoxazole and the cyclo­hexa-1,3-diene rings while the isoxazole ring is oriented at an acute angle of 63.46 (7)° with respect to the cyclo­hexa-1,3-diene ring. In the crystal, mol­ecules associate *via* C—H⋯N hydrogen bonds and C—H⋯π inter­actions, forming a three-dimensional network.

## Abstract 2

In the structure of the di­aza­dihydro­ace­naphthyl­ene derivative reported here, stabilization is provided by C—H⋯N and C—H⋯π inter­actions.

## Graphical abstract

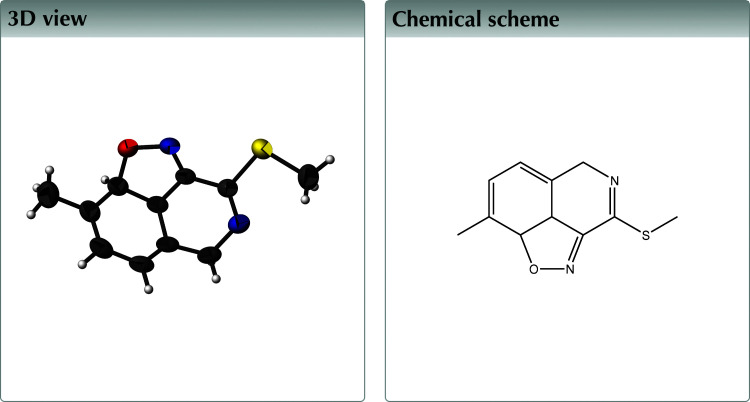

## Structure description

Di­aza­dihydro­ace­naphthyl­ene derivatives contain an isoxazoline scaffold and constitute an important class of heterocyclic compounds whose chemical properties have been investigated over the years (Jäger & Buss, 1980 ▸; Jäger *et al.*, 1980 ▸). This scaffold is used in the synthesis of several complex natural products (Saha & Bhattacharjya, 1997 ▸; Copp *et al.*,1992 ▸) and is a pharmacophore of numerous medicinal chemistry compounds (Brandi *et al.*, 2003 ▸; King *et al.*, 1982 ▸; Bacher *et al.*, 1997 ▸; You *et al.*, 1995 ▸). It has also been reported that this scaffold has a multiple range of biological activities, covering the agricultural field (Liu & Howe, 1983 ▸), medicinal properties such as anti­cancer, anti­biotic (Habeeb *et al.*, 2001 ▸; Mallesha *et al.*, 2001 ▸), anti­viral and anti-HIV (Ichiba *et al.*, 1993 ▸) agents.

**Figure 1:**
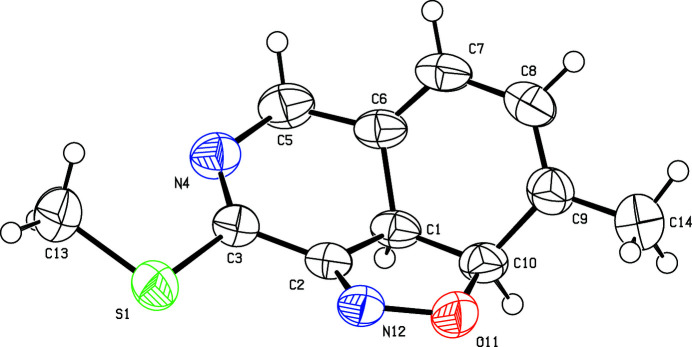
The mol­ecular structure of the title compound and the atomic numbering scheme. Displacement ellipsoids are drawn at the 50% probability level. H atoms are shown as spheres of arbitrary radius.

We report herein the synthesis and crystal structure of the title compound (Fig. 1 ▸). The 2,3,4,5-tetra­hydro-pyridine ring adopts a half-chair conformation with puckering parameters (Cremer & Pople, 1975 ▸) *Q*_T_ = 0.4779 (15) Å, θ = 129.65 (18)°, φ = 29.0 (2)° and is oriented at dihedral angles of 27.72 (7) and 45.17 (7)°, respectively, with the isoxazole and the cyclo­hexa-1,3-diene rings while the isoxazole ring makes an acute angle of 63.46 (7)° with respect to the cyclo­hexa-1,3-diene ring. These dihedral angles show that this tricycle compound is not planar, as confirmed by the total puckering amplitude *Q*_T_ of 1.4727 (15) Å.

**Figure 2:**
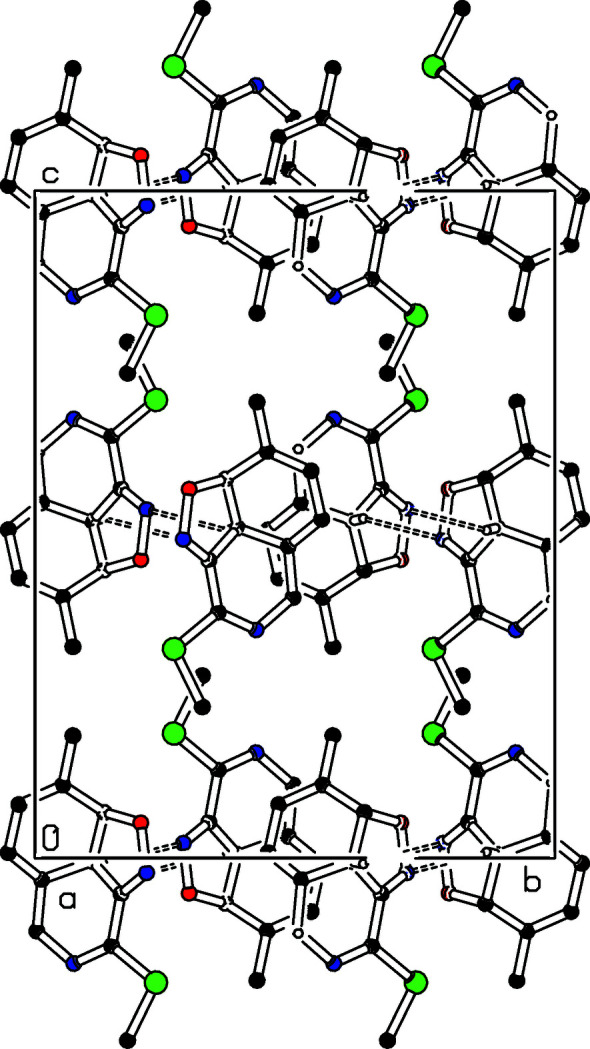
Part of the crystal packing of the title compound showing the formation of inter­molecular C1—H1⋯N12 hydrogen bonds along the **b** axis. Dashed lines indicate hydrogen bond contacts. H atoms not involved in hydrogen bond inter­actions have been omitted for clarity.

**Figure 3:**
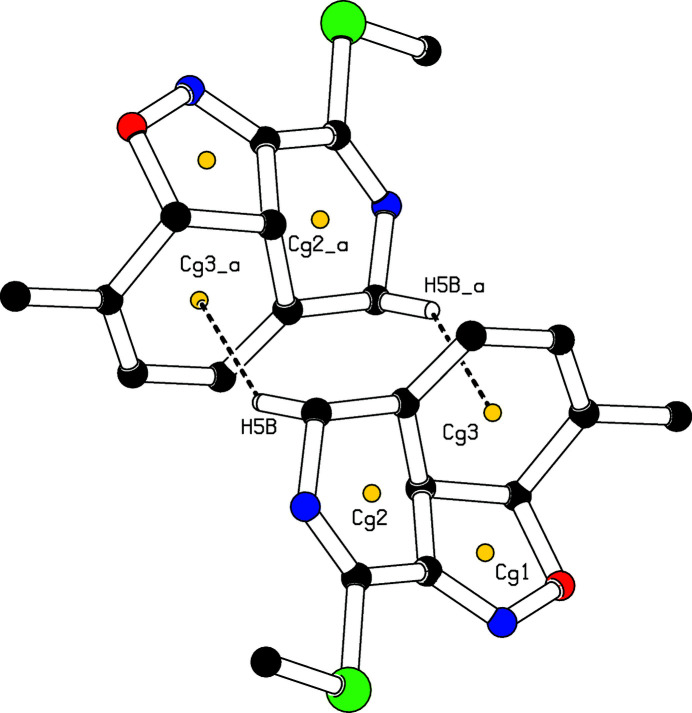
A view of the crystal packing, showing the π–ring inter­actions (dashed lines). The yellow dots are centroids of rings. H atoms not involved in these inter­actions have been omitted for clarity.

In the crystal, C1—H1⋯N12(*x* − 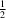 , −*y* + 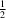 , −*z* + 2) hydrogen bonds (Table 1 ▸) link the mol­ecules along the [010] direction (Fig. 2 ▸) and C5—H5*B*⋯*Cg*3(−*x*, −*y* + 1, −*z*) inter­actions, where *Cg*3 is the centroid of the cyclo­hexa-1,3-diene ring (Fig. 3 ▸) are observed.

## Synthesis and crystallization

1-[(4-Methyl­benz­yl)amino]-1-methyl­thio-2-nitro­ethyl­ene (236 mg; 1 mmol) was dissolved in 4.4 ml (50 mmol) of triflic acid at a temperature within the range −26 to −15°C under a nitrogen atmosphere. The reaction was monitored as follows: one or two drops of the reacting medium were quenched over ice (about 1 g) and extracted with CH_2_Cl2_2_ (0.5 ml). The organic extract was dried over Na_2_CO_3_ and was purified by flash chromatography on a silica column (eluent: petroleum ether/ethyl acetate: (85:15, *v*/*v*) to afford the title compound (97 mg; 0.441 mmol) as a colourless powder. The powder was dissolved in a minimum of di­chloro­methane by heating under agitation. To this hot mixture, petroleum ether was added until the formation of a new precipitate started, which dissolved in the resulting mixture upon heating. Upon cooling, colourless crystals suitable for single-crystal X-ray diffraction analysis were obtained, m.p. 120.1°C.

**^1^H NMR (CDCl_3_)**: δ (p.p.m.) = 2.01 (*s*, 3 H, CH_3_); 2.39 (s, 3H, SCH_3_); 4.23 (*d*, *J* = 14.94 Hz, 1 H, H-8 b); 4.47 (*s*, 2 H, CH_2_); 5.46 (*d*, *J* = 14.9 Hz, 1 H, H-8a); 5.81 (*d*, J = 7.1 Hz, 1 H, vinylic H); 5.84 (*d*, *J* = 7.1 Hz, 1 H, vinylic H).

**^13^C NMR (CDCl_3_)**: δ (p.p.m.) = 12.0 (SCH_3_); 21.1 (CH_3_); 48.0 (C-8a); 58.4 (CH_2_); 82.0 (C-8 b); 117.8 (CH); 121.7 (CH); 124.8 (quaternary carbon); 130.4 (C-8); 152.6 (>C=N—O–); 155.4 (–S—C=N–).

**MS** (mass spectrometer, 70 eV); *m/z* (%): 220 [*M* ^+^]. **MS–HR(IE)** *m*/*z* ([*M* ^+^]) C_11_H_12_N_2_OS: 220.0680.

## Refinement

Crystal data, data collection and structure refinement details are summarized in Table 2 ▸.

## Supplementary Material

Crystal structure: contains datablock(s) I. DOI: 10.1107/S241431462100674X/xu4044sup1.cif

Structure factors: contains datablock(s) I. DOI: 10.1107/S241431462100674X/xu4044Isup2.hkl

Supporting information file. DOI: 10.1107/S241431462100674X/xu4044Isup3.cml

CCDC reference: 2089097

Additional supporting information:  crystallographic information; 3D view; checkCIF report

## Figures and Tables

**Table 1:** Hydrogen-bond geometry (Å, °) *Cg* is the centroid of the cyclo­hexa-1,3-diene ring.

*D*—H⋯*A*	*D*—H	H⋯*A*	*D*⋯*A*	*D*—H⋯*A*
C1—H1⋯N12^i^	0.98	2.64	3.4173 (16)	136
C5—H5*B*⋯*Cg*3^ii^	0.97	2.80	3.6158 (17)	142

Symmetry codes: (i) 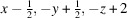 ; (ii) 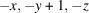 .

**Table 2:** Experimental details

Crystal data	
Chemical formula	C_11_H_12_N_2_OS
*M*_r_	220.29
Crystal system, space group	Orthorhombic, *P* *b* *c* *a*
Temperature (K)	293
*a*, *b*, *c* (Å)	8.0175 (2), 14.4611 (3), 18.5612 (4)
*V* (Å^3^)	2152.02 (8)
*Z*	8
Radiation type	Mo *K*α
μ (mm^−1^)	0.27
Crystal size (mm)	0.30 × 0.10 × 0.06

Data collection	
Diffractometer	Bruker CCD area detector
Absorption correction	Multi-scan (*SADABS*; Krause *et al.*, 2015 ▸)
*T*_min_, *T*_max_	0.922, 0.984
No. of measured, independent and observed [*I* > 2σ(*I*)] reflections	67637, 3155, 2560
*R*_int_	0.032
(sin θ/λ)_max_ (Å^−1^)	0.705

Refinement	
*R*[*F* ^2^ > 2σ(*F* ^2^)], *wR*(*F* ^2^), *S*	0.041, 0.129, 1.08
No. of reflections	3155
No. of parameters	138
H-atom treatment	H-atom parameters constrained
Δρ_max_, Δρ_min_ (e Å^−3^)	0.32, −0.19

Computer programs: *APEX3* (Bruker, 2018 ▸), *SAINT* (Bruker, 2016 ▸), *SIR2019* (Burla *et al.*, 2015 ▸), *PLATON* (Spek, 2020 ▸), *WinGX* (Farrugia, 2012 ▸), *SHELXL2018/3* (Sheldrick, 2015 ▸) and *publCIF* (Westrip, 2010 ▸).

## full crystallographic data

### Crystal data

**Table:**

C_11_H_12_N_2_OS	*D*_x_ = 1.360 Mg m^−^^3^
*M**_r_* = 220.29	Melting point: 393.1 K
Orthorhombic, *P**b**c**a*	Mo *K*α radiation, λ = 0.71073 Å
Hall symbol: -P 2ac 2ab	Cell parameters from 8401 reflections
*a* = 8.0175 (2) Å	θ = 2.2–29.8°
*b* = 14.4611 (3) Å	µ = 0.27 mm^−^^1^
*c* = 18.5612 (4) Å	*T* = 293 K
*V* = 2152.02 (8) Å^3^	Parallelepiped, colorless
*Z* = 8	0.30 × 0.10 × 0.06 mm
*F*(000) = 928	

### Data collection

**Table:**

Bruker CCD area detector diffractometer	3155 independent reflections
Radiation source: fine-focus sealed tube	2560 reflections with *I* > 2σ(*I*)
Graphite monochromator	*R*_int_ = 0.032
Detector resolution: 512 pixels mm^-1^	θ_max_ = 30.1°, θ_min_ = 2.8°
phi and ω scans	*h* = −11→11
Absorption correction: multi-scan (SADABS; Krause *et al.*, 2015)	*k* = −20→20
*T*_min_ = 0.922, *T*_max_ = 0.984	*l* = −26→26
67637 measured reflections	

### Refinement

**Table:**

Refinement on *F*^2^	Primary atom site location: structure-invariant direct methods
Least-squares matrix: full	Secondary atom site location: difference Fourier map
*R*[*F*^2^ > 2σ(*F*^2^)] = 0.041	Hydrogen site location: inferred from neighbouring sites
*wR*(*F*^2^) = 0.129	H-atom parameters constrained
*S* = 1.08	*w* = 1/[σ^2^(*F*_o_^2^) + (0.0606*P*)^2^ + 0.5724*P*] where *P* = (*F*_o_^2^ + 2*F*_c_^2^)/3
3155 reflections	(Δ/σ)_max_ = 0.001
138 parameters	Δρ_max_ = 0.32 e Å^−^^3^
0 restraints	Δρ_min_ = −0.19 e Å^−^^3^
0 constraints	

### Special details

**Table:**

Geometry. All esds (except the esd in the dihedral angle between two l.s. planes) are estimated using the full covariance matrix. The cell esds are taken into account individually in the estimation of esds in distances, angles and torsion angles; correlations between esds in cell parameters are only used when they are defined by crystal symmetry. An approximate (isotropic) treatment of cell esds is used for estimating esds involving l.s. planes.

### Fractional atomic coordinates and isotropic or equivalent isotropic displacement parameters (Å^2^)

**Table:**

	*x*	*y*	*z*	*U*_iso_*/*U*_eq_	
S1	0.87930 (6)	0.23268 (3)	0.81332 (2)	0.05945 (15)	
C1	0.68279 (16)	0.11024 (8)	0.99475 (8)	0.0430 (3)	
H1	0.567035	0.131511	0.991152	0.052*	
C2	0.79398 (16)	0.16562 (8)	0.94630 (7)	0.0411 (3)	
C3	0.78747 (16)	0.14749 (9)	0.86830 (8)	0.0441 (3)	
N4	0.72257 (16)	0.07471 (9)	0.84198 (7)	0.0527 (3)	
C5	0.6511 (2)	0.00367 (11)	0.89045 (9)	0.0581 (4)	
H5A	0.685682	−0.056803	0.873466	0.070*	
H5B	0.530492	0.006443	0.887046	0.070*	
C6	0.69925 (16)	0.01268 (9)	0.96758 (8)	0.0458 (3)	
C7	0.77226 (18)	−0.05134 (9)	1.00803 (9)	0.0512 (3)	
H7	0.786148	−0.110657	0.989631	0.061*	
C8	0.8308 (2)	−0.03094 (10)	1.08014 (9)	0.0543 (4)	
H8	0.871921	−0.079538	1.107742	0.065*	
C9	0.8293 (2)	0.05336 (11)	1.10934 (9)	0.0544 (3)	
C10	0.7554 (2)	0.13333 (9)	1.06827 (8)	0.0492 (3)	
H10	0.668392	0.162125	1.097811	0.059*	
O11	0.88721 (16)	0.20355 (7)	1.05266 (6)	0.0584 (3)	
N12	0.90369 (16)	0.21466 (8)	0.97783 (7)	0.0486 (3)	
C13	0.8627 (3)	0.17816 (14)	0.72661 (9)	0.0685 (5)	
H13A	0.748115	0.177828	0.711577	0.103*	
H13B	0.902781	0.115738	0.729744	0.103*	
H13C	0.928286	0.211794	0.692175	0.103*	
C14	0.8944 (4)	0.07297 (16)	1.18338 (10)	0.0846 (7)	
H14A	0.928090	0.016115	1.205768	0.127*	
H14B	0.808424	0.101512	1.211680	0.127*	
H14C	0.988454	0.113844	1.180153	0.127*	

### Atomic displacement parameters (Å^2^)

**Table:**

	*U*^11^	*U*^22^	*U*^33^	*U*^12^	*U*^13^	*U*^23^
S1	0.0740 (3)	0.0518 (2)	0.0525 (2)	−0.00919 (18)	0.00304 (17)	0.00618 (15)
C1	0.0366 (6)	0.0335 (5)	0.0588 (7)	0.0038 (4)	0.0087 (5)	0.0000 (5)
C2	0.0417 (6)	0.0302 (5)	0.0513 (7)	0.0016 (4)	0.0033 (5)	−0.0005 (5)
C3	0.0415 (6)	0.0407 (6)	0.0502 (7)	0.0004 (5)	−0.0010 (5)	0.0000 (5)
N4	0.0512 (6)	0.0494 (6)	0.0576 (7)	−0.0054 (5)	−0.0049 (5)	−0.0056 (5)
C5	0.0584 (8)	0.0449 (7)	0.0710 (10)	−0.0145 (7)	−0.0018 (7)	−0.0085 (7)
C6	0.0394 (6)	0.0339 (6)	0.0641 (8)	−0.0058 (5)	0.0093 (6)	−0.0037 (5)
C7	0.0477 (7)	0.0319 (5)	0.0739 (9)	−0.0012 (5)	0.0151 (7)	−0.0008 (6)
C8	0.0523 (8)	0.0436 (7)	0.0669 (9)	0.0072 (6)	0.0129 (7)	0.0123 (6)
C9	0.0590 (8)	0.0511 (8)	0.0530 (8)	0.0045 (7)	0.0112 (6)	0.0062 (6)
C10	0.0542 (7)	0.0393 (6)	0.0540 (7)	0.0051 (6)	0.0137 (6)	−0.0016 (5)
O11	0.0802 (8)	0.0458 (5)	0.0493 (6)	−0.0139 (5)	−0.0003 (5)	−0.0045 (4)
N12	0.0596 (7)	0.0358 (5)	0.0504 (6)	−0.0081 (5)	0.0009 (5)	−0.0014 (4)
C13	0.0823 (12)	0.0727 (11)	0.0507 (8)	0.0071 (9)	0.0068 (8)	−0.0005 (8)
C14	0.123 (2)	0.0730 (12)	0.0575 (10)	0.0118 (12)	−0.0060 (11)	0.0052 (9)

### Geometric parameters (Å, º)

**Table:**

S1—C3	1.7610 (14)	C7—H7	0.9300
S1—C13	1.7972 (18)	C8—C9	1.334 (2)
C1—C2	1.4984 (18)	C8—H8	0.9300
C1—C6	1.5039 (17)	C9—C14	1.497 (3)
C1—C10	1.521 (2)	C9—C10	1.506 (2)
C1—H1	0.9800	C10—O11	1.4942 (18)
C2—N12	1.2724 (17)	C10—H10	0.9800
C2—C3	1.4723 (19)	O11—N12	1.4045 (16)
C3—N4	1.2717 (17)	C13—H13A	0.9600
N4—C5	1.481 (2)	C13—H13B	0.9600
C5—C6	1.489 (2)	C13—H13C	0.9600
C5—H5A	0.9700	C14—H14A	0.9600
C5—H5B	0.9700	C14—H14B	0.9600
C6—C7	1.328 (2)	C14—H14C	0.9600
C7—C8	1.449 (2)		
			
C3—S1—C13	100.43 (8)	C9—C8—C7	123.94 (14)
C2—C1—C6	104.35 (10)	C9—C8—H8	118.0
C2—C1—C10	101.16 (11)	C7—C8—H8	118.0
C6—C1—C10	118.25 (12)	C8—C9—C14	122.87 (16)
C2—C1—H1	110.8	C8—C9—C10	119.97 (15)
C6—C1—H1	110.8	C14—C9—C10	117.15 (15)
C10—C1—H1	110.8	O11—C10—C9	109.97 (13)
N12—C2—C3	125.17 (12)	O11—C10—C1	104.24 (10)
N12—C2—C1	115.67 (12)	C9—C10—C1	115.85 (12)
C3—C2—C1	118.29 (11)	O11—C10—H10	108.8
N4—C3—C2	122.66 (13)	C9—C10—H10	108.8
N4—C3—S1	121.83 (12)	C1—C10—H10	108.8
C2—C3—S1	115.49 (9)	N12—O11—C10	109.64 (10)
C3—N4—C5	119.94 (13)	C2—N12—O11	109.03 (11)
N4—C5—C6	115.04 (12)	S1—C13—H13A	109.5
N4—C5—H5A	108.5	S1—C13—H13B	109.5
C6—C5—H5A	108.5	H13A—C13—H13B	109.5
N4—C5—H5B	108.5	S1—C13—H13C	109.5
C6—C5—H5B	108.5	H13A—C13—H13C	109.5
H5A—C5—H5B	107.5	H13B—C13—H13C	109.5
C7—C6—C5	126.65 (14)	C9—C14—H14A	109.5
C7—C6—C1	120.20 (14)	C9—C14—H14B	109.5
C5—C6—C1	112.45 (13)	H14A—C14—H14B	109.5
C6—C7—C8	121.55 (13)	C9—C14—H14C	109.5
C6—C7—H7	119.2	H14A—C14—H14C	109.5
C8—C7—H7	119.2	H14B—C14—H14C	109.5

### Hydrogen-bond geometry (Å, º)

*Cg* is the centroid of the cyclohexa-1,3-diene ring.

**Table:**

*D*—H···*A*	*D*—H	H···*A*	*D*···*A*	*D*—H···*A*
C1—H1···N12^i^	0.98	2.64	3.4173 (16)	136
C5—H5*B*···*Cg*3^ii^	0.97	2.80	3.6158 (17)	142

Symmetry codes: (i) *x*−1/2, −*y*+1/2, −*z*+2; (ii) −*x*, −*y*+1, −*z*.
